# Predicting value of five anthropometric measures in metabolic syndrome among Jiangsu Province, China

**DOI:** 10.1186/s12889-020-09423-9

**Published:** 2020-08-31

**Authors:** Ting Tian, Jingxian Zhang, Qianrang Zhu, Wei Xie, Yuanyuan Wang, Yue Dai

**Affiliations:** Institute of Food Safety and Assessment, Jiangsu Provincial Center for Disease Control and Prevention, No.172 Jiangsu Road, Nanjing, 210009 Jiangsu China

**Keywords:** Metabolic syndrome, Waist circumference, WHtR, ABSI, BRI

## Abstract

**Background:**

Metabolic syndrome (MetS), a condition of metabolic disorders, is now causing large disease burden around the world. This study aimed to update the prevalence of MetS in Jiangsu Province of China and evaluate the predicting value of five anthropometric measures including waist circumference (WC), body mass index (BMI), waist-to-height ratio (WHtR), a body shape index (ABSI) and body roundness index (BRI) in MetS.

**Methods:**

8040 participants from 12 survey sites were enrolled into this cross-sectional study by multi-stage stratified cluster random sampling method from 2014 nutrition and diet investigation project in Jiangsu Province. The transformation of sex-specific z-score made the comparison meaningful when conducting the logistic analysis between anthropometric indices and MetS. The abilities of anthropometric indices to predict MetS were evaluated by the receiver operating characteristic curve (ROC). *Delong* test was applied to compare area under different ROC curves.

**Results:**

The prevalence of MetS in Jiangsu Province was 35.2% and the standardized prevalence was 34.8%. WC, BMI, WHtR, ABSI and BRI z-scores were positively related to MetS and its components. WC, WHtR and BRI z-score had stronger associations with MetS than BMI and ABSI in both male and female population. WC, WHtR and BRI had larger area under ROC curve than BMI and ABSI in male and female. WC in men had the largest area under the ROC curve, significantly higher than the other four measures of BMI, WHtR, ABSI and BRI (Z value = 9.08, 2.88, 16.73, 2.75 respectively). Among women, WC, WHtR and BRI had larger area under the ROC curve than BMI and ABSI, but the area under the WC, WHtR and BRI curve were not significantly different after the pairwise comparison by the *Delong* test. The optimal cut-off values of WC, WHtR and BRI for predicting MetS was 85.25 cm, 0.52 and 3.61 in male, 80.05 cm, 0.51 and 3.83 in female.

**Conclusion:**

MetS has become one of the major chronic diseases in Jiangsu Province. WC was better than other four indices in predicting MetS among male population in Jiangsu. WC, WHtR and BRI had superior abilities than BMI/ABSI in predicting MetS among female population.

## Background

Metabolic syndrome (MetS) is a group of metabolic disorders including obesity, elevated blood glucose, dyslipidemia and high blood pressure [1]. Additional energy intake and sedentary lifestyle caused the accumulation of central and visceral fats, further developing the abnormity of metabolism [2]. Furthermore, MetS is a crucial multiplex risk factor of cardiovascular diseases and type 2 diabetes [3]. Results from a study demonstrates that patients with MetS have a two-fold increased risk of cardiovascular disease and a five-fold increased risk of type 2 diabetes [4].

MetS is at a high prevalence level around the world. United States National Health and Nutrition Survey revealed that more than one-third US adults had MetS [3]. The trend of MetS in developing countries is also gradually rising. According to Chinese national surveillance data in 2010, the prevalence of MetS among adults in mainland China were 33.9%. As for Jiangsu Province in eastern China, the most recent prevalence of MetS was 32.7% surveyed in 2010. With the great changes of lifestyle and dietary habits, the update of MetS condition in this region is urgently needed.

Early identification and intervention of at-risk subjects can prevent the ongoing of MetS and control the progression to other chronic diseases, which will bring lots of benefits to human health and reduce the individual and societal burden of related diseases. In view of the above reasons, there are a rising number of anthropometric indicators applied to predict MetS. This study chose non-invasive, low-cost and easily-calculated measures, including waist circumference (WC), body mass index (BMI), waist-to-height ratio (WHtR) [5], a body shape index (ABSI) [6], and body roundness index (BRI) [7] and compared their abilities in predicting MetS.

Obesity condition was represented by WC and BMI. WC is a simple, inexpensive method to measure central obesity [8]. WHtR has been shown to be associated with obesity-related diseases [9]. The use of WHtR as a better predictor of CVD risk factor has been supported by Meta-analysis [10]. ABSI invented by Krakauer et al. was firstly used in the prediction of death risk [6]. A related research illuminated that there was significant association between ABSI and central obesity, besides, it was better than BMI in the prediction of type 2 diabetes [11]. BRI was created by Thomas et al. for measuring the association with visceral fat accumulation at the first time [7]. The study in Peruvian adults found that BRI had clinical diagnostic value in predicting MetS [12]. As time went on, the application of these indices has been further extended and researchers have exerted tremendous fascination on exploring the association between these indicators and MetS components. It seems that using these widely-used and easily-calculated anthropometric measures are promising in predicting MetS.

Up to now, there are some relevant researches comparing the predicting value of several anthropometric indicators in MetS among Chinese population, however, the study subjects are either from a single center [5] or a small sample size [13]. Therefore, this study selected regional representative samples through scientific sampling methods, aiming to update the prevalence of MetS and its components in Jiangsu Province and find out the potential regional representative anthropometric indicators in predicting MetS among adults based on 2014 nutrition and diet investigation project in Jiangsu Province.

## Methods

### Participants

This was a cross-sectional study consistent with checklist of items in STROBE statement (seen in Additional file 1) and the investigation was based on the 2014 nutrition and diet investigation project in Jiangsu Province of eastern China. Participants were recruited representatively by multi-stage stratified cluster sample method from September to November in 2014. In the first stage, by the method of systematic sampling, the survey counties (districts) were selected as the survey sites in rural or urban areas. In the whole province, 12 areas, Jiangyin, Changshu, Taicang, Jurong, Jianye, Qinhuai, Haimen, Dafeng, Sihong, Quanshan, Suining and Tongshan, were selected as the survey sites. In the second stage, 3 townships/streets (a city administrative area at the same level as the township in China) were randomly chosen at each survey site. In the third stage, 2 villages/neighborhood committee were randomly selected at each township/street. In the fourth stage, 75 households were randomly selected from each village/neighborhood committee. All the adult family members in the household, who aged over 18 years old, were enrolled in this investigation. If the participants refused to be investigated, then the corresponding number of substitutes were chosen at the same survey site. A total of 8805 individuals were investigated, 765 individuals were excluded because of missing key information, and finally 8040 individuals were included. Smoking/Drinking was defined as those who were existing smoke/drink habits at the survey time.

### Data collection

Epidemiological survey: The structured questionnaire was administered to collect the basic information (age, gender, education level, smoking history and drinking history). Hypertension, diabetes, dyslipidemia and family history of the respondents were interviewed by the unified trained investigators.

Physical examination: After the interview, the height, weight, waist circumference and blood pressure were measured by the investigators. Height was measured once in centimeter (cm); weight was measured once in kilogram (kg). Waist circumference and blood pressure were measured in a standard protocol [14]. When measuring WC by trained investigators, the participants were required to stand upright, relax their abdomen, naturally droop their arms, and put their feet together (both legs were loaded). The measuring point of WC was at the middle point between the bottom of the rib cage and the uppermost border of the iliac crests at the end of exhalation in standing positions with an inelastic tape [11]. The soft ruler was moderately tightened, then mark the measuring points on both sides, repeat the measurement twice, and record the average value in precision of 0.1 cm. Blood pressure (BP) was measured three times. BP was measured by the standard mercury sphygmomanometer and the unit was millimeter mercury (mmHg). After 5 min’ sedentary seat, first measurement was taken by the trained investigators. In total, three measurements were performed, and one minute between each measurement. The three measurements were averaged as the blood pressure values of the individual. Kept one decimal place for all measurement results.

Laboratory examination: 5 ml of fasting venous blood was collected in the morning to measure level of triglyceride, high-density lipoprotein and fasting plasma glucose (FPG).

Strict quality control was implemented throughout the investigation and laboratory examination.

### Diagnostic criteria for MetS

The diagnostic criteria for the MetS and its components in the Chinese population were based on the 2009 Joint Interim Statement (JIS) [15], (1) Central obesity: male/female’s waist circumference ≥ 85/80 cm; (2) Elevated triglycerides: TG > 1.70 mmol/L or those who received drug treatment for hypertriglyceridemia; (3) Reduced HDL-C: male/female’s HDL-C < 1.0 mmol/L/1.30 mmol/L or those who treated for reduced HDL-C; (4) Elevated blood pressure: systolic blood pressure (SBP) / diastolic blood pressure (DBP) ≥130/85 mmHg or those who were treated with hypotensive therapy; (5) Elevated fasting glucose: FPG ≥ 5.6 mmol/L or those who received anti-hyperglycemia treatment. According to the above criteria, those who met the three and above criteria were diagnosed as MetS.

### Calculation formula for anthropometric indicators

BMI, WHtR, ABSI and BRI were used in this study, and the calculation formulas were shown as below.

1. $$ BMI=\frac{weight}{height^2} $$
2. $$ \mathrm{WHtR}=\frac{WC}{height} $$
3. $$ ABSI=\frac{WC}{\left({height}^{\frac{1}{2}}\times {BMI}^{\frac{2}{3}}\right)} $$
4. $$ BRI=364.2-365.5\sqrt{1-\frac{{\left(\frac{WC}{2\pi}\right)}^2}{{\left(0.5\times height\right)}^2}} $$

### Statistical analysis

Epidata3.01 software was used to input the questionnaire and laboratory data. SPSS (statistical product and service solutions) version 23.0 software of IBM Company was applied to analyze the data. The continuous variables in accordance with the normal distribution were expressed by mean ± standard deviation, and the quantitative data were compared by independent sample *t*-test. The standardized prevalence of MetS and its components was adjusted in the following formula, using the data of the sixth Chinese national population census. The WC was converted to a z-score by this equation: (WC-WC_mean_)/WC_SD_. BMI, WHtR, ABSI and BRI were also transformed to z-scores by the same equation. The association between each z-score and MetS was measured by the logistic analysis, and adjusted factors were age, gender, drinking and smoking conditions. Sex-specific z-score of these indicators would be transformed when conducting sex stratification logistic analysis, and the adjusted factors were age, drinking and smoking conditions. The z-score made the comparison meaningful because the unit change in the logistic regression analysis was different for the chosen anthropometric measures. The area under the receiver operating characteristic curve (AUROC) was the measurement to estimate the ability of each anthropometric indicator in predicting MetS, and the optimal value of each indicator was determined by the Youden’s index. *Delong* test in the pROC package of R software version 3.6.1 was used to compare the area under different curves. *P* value < 0.05 was considered statistical significance.

$$ standed\ prevelance=\frac{Number\ of\ Chinese\ male\ast Male\ prevalence\ in\ Jiangsu+ Number\ of\ Chinese\ female\ast Female\ prevalence\ in\ Jiangsu}{Total\ population\ number\ of\ China} $$

## Results

### General information of participants

In this study, 8040 individuals were enrolled, including 3606 men (44.9%) and 4434 women (55.1%). As shown in Table 1, a significant difference was existed in education level between men and women. The average waist circumference of men was 86.17 cm, which was higher than that of women’s 81.71 cm (*P* < 0.001). Average of WHtR in men was lower than in women, 0.51 to 0.52 (*P* < 0.001). The mean value of BMI was even between men and women. The proportion of smoking and drinking in men was higher than that in women (smoking: male 49.9% vs. female 7.2%, drinking: male 49.4% vs. 7.2%).

**Table 1:** Basic information of research population

Variables	Male (%)	Female (%)	*P* value
	*N* = 3606	*N* = 4434	
Age	54.73 ± 15.12	53.73 ± 15.09	0.003
≤ 54	1639 (45.5)	2205 (49.7)	<0.001
≥ 55	1967 (54.5)	2229 (50.3)	
Education level^a^			<0.001
Primary school and below	280 (7.8)	1019 (23.0)	
Middle school	2439 (67.7)	2670 (60.3)	
University and above	885 (24.6)	737 (16.7)	
WC (cm)	86.17 ± 16.23	81.71 ± 10.19	<0.001
BMI (kg/m^2^)	24.31 ± 4.91	24.27 ± 3.97	0.650
WHtR	0.51 ± 0.059	0.52 ± 0.067	<0.001
Smoking			<0.001
no	1801 (50.1)	4326 (97.9)	
yes	1795 (49.9)	95 (2.1)	
Drinking			<0.001
no	1825 (50.6)	4115 (92.8)	
yes	1781 (49.4)	319 (7.2)	
ABSI	7.95 ± 0.66	7.83 ± 0.68	<0.001
BRI	3.67 ± 1.16	3.89 ± 1.36	<0.001

^a^Education level is not of all subjects

*WC* Waist circumference, *BMI* body mass index, *WHtR* waist-to-height ratio, *ABSI* a body shape index, *BRI* body roundness index

### Prevalence of MetS and its components in adult residents of Jiangsu Province

**Fig. 1:**
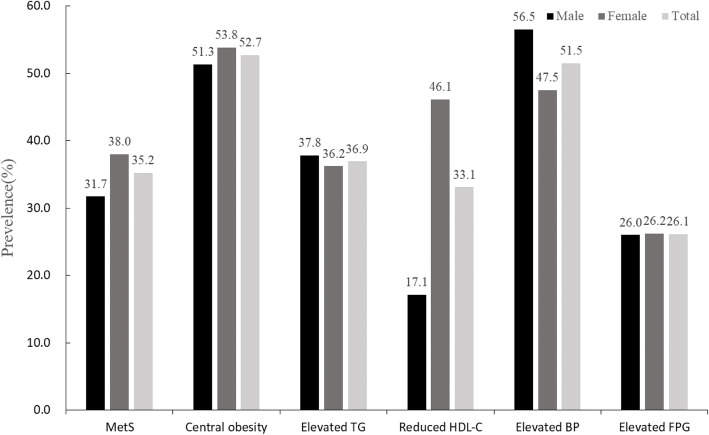
Prevalence of MetS and its components in Jiangsu adult residents. Central obesity: male/female’s waist circumference ≥ 85/80 cm

The prevalence of MetS was 35.2% in Jiangsu Province, and 34.8% after adjusted by information from the sixth Chinese national population census. The proportion of central obesity (male/female WC > 85/80 cm) was 52.7%, elevated triglycerides (> 1.70 mmol/L) was 36.9%, reduced HDL-C (male/female HDL-C < 1.0/1.30 mmol/L) was 33.1%, elevated blood pressure (≥130/85 mmHg) was 51.5%, and elevated fasting glucose (FPG ≥ 5.6 mmol/L) was 26.1%. The prevalence of MetS in women was 38.0%, higher than 31.7% in men. Women were prone to have central obesity with a prevalence of 53.8% than men, which was 51.3%. The prevalence of reduced HDL-C in women was 46.1%, significantly higher than 17.1% in men, while the prevalence of elevated blood pressure was 47.5%, lower than 56.5% in men. (For more details displayed in Fig. 1 and Table 2).

**Table 2:** Prevalence of MetS and its components in Jiangsu adult residents

MetS components	Male (%)	Female (%)	*P* value	Crude/standardized prevalence (%)
MetS	31.7	38.0	<0.001	35.2/34.8
Central obesity	51.3	53.8	0.027	52.7/52.5
Elevated triglycerides	37.8	36.2	0.138	36.9/37.0
Reduced HDL-C	17.1	46.1	<0.001	33.1/31.2
Elevated blood pressure	56.5	47.5	<0.001	51.5/52.1
Elevated fasting glucose	26.0	26.2	0.835	26.1/26.1

MetS: metabolic syndrome; Central obesity: male/female’s waist circumference ≥ 85/80 cm

### Associations of five anthropometric measures with MetS and its components

Table 3 demonstrated the associations of five anthropometric measures with MetS and its components. In the total population, after adjusted by age, gender, drinking, and smoking conditions, z-scores of WC, BMI, WHtR, ABSI and BRI were positively associated with MetS and most of its components (adjusted OR > 1), while the association intensity of WC, WHtR and BRI with MetS was higher than that of BMI and ABSI. Sex stratification logistic analysis indicated that WC, WHtR and BRI sex-specific z-scores still had strong associations with MetS (all adjusted OR > 1) in both male and female population after adjusting age, drinking and smoking conditions. In male population, WHtR z-score had the highest odds ratio in predicting Mets. If WHtR z-score increases by one unit, the risk of MetS would be affected by 3.82 times. The WC z-score increased by one unit, and the risk of MetS increased by 3.54 times in female. There were stronger associations between WC/WHtR/BRI z-score and elevated triglycerides and reduced HDL-C in both genders. BMI z-score had higher associations with elevated blood pressure and elevated fasting glucose in both genders.

**Table 3:** Associations of five anthropometric measures with MetS and its components

MetS components	WC z-Score	BMI z-Score	WHtR z-Score	ABSI z-Score	BRI z-Score
MetS					
Adjusted OR	**3.66 (3.42 ~ 3.92)**	2.74 (2.57 ~ 2.92)	3.38 (3.16 ~ 3.61)	1.63 (1.55 ~ 1.72)	3.27 (3.06 ~ 3.50)
Male adjusted OR	3.79 (3.41 ~ 4.22)	3.02 (2.73 ~ 3.35)	**3.82 (3.42 ~ 4.27)**	1.71 (1.58 ~ 1.86)	3.76 (3.37 ~ 4.21)
Female adjusted OR	**3.54 (3.23 ~ 3.88)**	2.55 (2.36 ~ 2.76)	3.10 (2.85 ~ 3.37)	1.56 (1.45 ~ 1.67)	2.98 (2.74 ~ 3.24)
Elevated triglycerides					
Adjusted OR	**1.52 (1.45 ~ 1.60)**	1.36 (1.29 ~ 1.43)	1.47 (1.40 ~ 1.54)	1.23 (1.17 ~ 1.29)	1.46 (1.39 ~ 1.53)
Male adjusted OR	1.67 (1.56 ~ 1.81)	1.51 (1.39 ~ 1.63)	1.69 (1.56 ~ 1.83)	1.33 (1.24 ~ 1.44)	**1.71 (1.58 ~ 1.86)**
Female adjusted OR	**1.37 (1.29 ~ 1.46)**	1.25 (1.18 ~ 1.33)	1.30 (1.22 ~ 1.39)	1.14 (1.07 ~ 1.21)	1.29 (1.21 ~ 1.37)
Reduced HDL-C					
Adjusted OR	1.59 (1.51 ~ 1.68)	1.49 (1.41 ~ 1.57)	**1.73 (1.61 ~ 1.85)**	1.15 (1.10 ~ 1.21)	1.51 (1.44 ~ 1.59)
Male adjusted OR	1.80 (1.64 ~ 1.97)	1.74 (1.58 ~ 1.92)	**1.83 (1.66 ~ 2.01)**	1.26 (1.15 ~ 1.38)	1.80 (1.64 ~ 1.98)
Female adjusted OR	**1.49 (1.39 ~ 1.59)**	1.39 (1.31 ~ 1.48)	1.40 (1.32 ~ 1.49)	1.10 (1.04 ~ 1.17)	1.39 (1.31 ~ 1.48)
Elevated blood pressure					
Adjusted OR	1.61 (1.53 ~ 1.70)	**1.77 (1.67 ~ 1.87)**	1.63 (1.55 ~ 1.72)	1.05 (0.99 ~ 1.10)	1.62 (1.54 ~ 1.71)
Male adjusted OR	1.52 (1.41 ~ 1.65)	**1.81 (1.66 ~ 1.99)**	1.57 (1.44 ~ 1.70)	1.01 (0.93 ~ 1.09)	1.56 (1.43 ~ 1.70)
Female adjusted OR	1.66 (1.54 ~ 1.79)	**1.73 (1.61 ~ 1.87)**	1.66 (1.55 ~ 1.78)	1.07 (1.00 ~ 1.14)	1.65 (1.53 ~ 1.77)
Elevated fasting glucose					
Adjusted OR	1.31 (1.24 ~ 1.38)	**1.35 (1.28 ~ 1.43)**	1.28 (1.22 ~ 1.36)	1.00 (0.95 ~ 1.06)	1.27 (1.21 ~ 1.34)
Male adjusted OR	1.28 (1.18 ~ 1.38)	**1.46 (1.34 ~ 1.59)**	1.30 (1.20 ~ 1.42)	0.94 (0.87 ~ 1.03)	1.30 (1.19 ~ 1.41)
Female adjusted OR	**1.33 (1.24 ~ 1.43)**	1.29 (1.21 ~ 1.38)	1.27 (1.19 ~ 1.36)	1.04 (0.97 ~ 1.12)	1.26 (1.18 ~ 1.34)

*OR* odds ratio, *WC* Waist circumference, *BMI* body mass index, *WHtR* waist-to-height ratio, *ABSI* a body shape index, *BRI* body roundness index, *MetS* metabolic syndrome

The anthropometric measures were converted to a z-score using this equation:(X-X_mean_)/X_SD_

Adjusted factors of whole population: age, gender (male/female), drinking (yes/no), smoking (yes/no);

Gender stratification adjusted factors: age, drinking (yes/no), smoking (yes/no)

### ROC curve of five anthropometric indices to predict MetS

In Table 4, the AUROC of different indicators for predicting the MetS in both male and female were compared. In male, the area under the curve of WC was 0.810 (95% CI = 0.795–0.824), and the area under the curve of WHtR and BRI was the same, which was 0.800 (95% CI = 0.785–0.815). In female, the area under the curve of WC was 0.801 (95% CI = 0.788–0.814), and the area under the curve of WHtR and BRI was the same, which was 0.802 (95% CI = 0.789–0.815). WC, WHtR and BRI had larger area under the ROC curve than BMI and ABSI in both genders after pairwise comparisons by the *Delong* test in supplementary Table S1 of Additional file 2. WC in men had the largest area under the ROC curve, the area under the WC curve was significantly higher than the other four indicators, BMI, WHtR, ABSI and BRI (*Z* value = 9.08, 2.88, 16.73, 2.75 respectively). Among women, WC, WHtR and BRI had the larger area under the ROC curve, which were 0.801 (95% CI = 0.788–0.814), 0.802(95% CI = 0.789–0.815) and 0.802(95% CI = 0.789–0.815), respectively. After the pairwise comparison of the *Delong* test, the area under the WC, WHtR and BRI were not significantly different.

**Table 4:** Area under the ROC (95%CI) for the anthropometric indicators and MetS

	WC	BMI	WHtR	ABSI	BRI
Whole population	0.787 (0.776–0.797)	0.736 (0.725–0.748)	0.803 (0.793–0.812)	0.656 (0.644–0.668)	0.803 (0.793–0.812)
Male	0.810 (0.795–0.824)	0.741 (0.724–0.758)	0.800 (0.785–0.815)	0.665 (0.646–0.683)	0.800 (0.785–0.815)
Female	0.801 (0.788–0.814)	0.735 (0.720–0.750)	0.802 (0.789–0.815)	0.660 (0.644–0.676)	0.802 (0.789–0.815)

*WC* Waist circumference, *BMI* body mass index, *WHtR* waist-to-height ratio, *ABSI* a body shape index, *BRI* body roundness index, *MetS* metabolic syndrome

Optimal cut-off values of WC, WHtR and BRI for predicting MetS were displayed in Table 5. In male, when the WC cut-off value was 85.25 cm, the maximum Youden’s index was 0.55. The sensitivity was 88.1% and the specificity was 66.6%. In female, when the WC cut-off value was 80.05, the maximum Youden’s index was 0.52. The sensitivity was 85.9% and the specificity was 65.9%. The optimal cut-off value of WHtR in male was 0.52 determined by the maximum Youden’s index (0.48). The sensitivity was 81.0% and the specificity was 66.9%. In female, when the WHtR cut-off value was 0.51, the maximum Youden’s index was 0.48. The sensitivity was 76.9% and the specificity was 71.3%. The optimal BRI cut-off value in male was 3.61 and the maximum Youden’s index was 0.48. The sensitivity was 81.0%, the specificity was 66.9%. In female, when the BRI cut-off value was 3.83, the maximum Youden’s index was 0.48, and the sensitivity was 76.9%, the specificity was 71.3%.

**Table 5:** Optimal cut-off points of WC, WHtR and BRI for predicting MetS

	Optimal cut-off points	Sensitivity (%)	Specificity (%)	Youden’s index
WC				
Male	85.25	88.1	66.6	0.55
Female	80.05	85.9	65.9	0.52
WHtR				
Male	0.52	81.0	66.9	0.48
Female	0.51	76.9	71.3	0.48
BRI				
Male	3.61	81.0	66.9	0.48
Female	3.83	76.9	71.3	0.48

*WC* Waist circumference, *WHtR* waist-to-height ratio, *BRI* body roundness index, *MetS* metabolic syndrome

## Discussions

This study updated the condition of MetS and its components among Jiangsu adult residents in eastern China through a well-designed study and evaluated the ability of five non-invasive, low-cost and easily-calculated anthropometric measures, including WC, BMI, WHtR, ABSI and BRI, in predicting MetS. It turned out that, Jiangsu were at high burden of Mets and its components, and WC/WHtR/BRI had good regional representative predicting value for MetS in Jiangsu Province.

We found that the prevalence of MetS among adult residents in Jiangsu Province was 35.2% in 2014. After standardized by the data from the sixth Chinese national census, the standardized prevalence was 34.8%, which was higher than 33.9% in 2010 in China [16]. Under the same 2009 JIS diagnostic criteria, the crude prevalence of MetS in Jiangsu adults in 2010 was 38.9%, and the standardized prevalence was 32.7% [17]. The prevalence of MetS in Jiangsu Province was basically stable over a four-year period. The prevalence of central obesity was 52.7% and 52.5% after standardization. This was close to the central obesity prevalence of rural adult in Chinese Gansu Province, which was 53.18% [18]. The JIS diagnostic criteria for central obesity (male/female’s WC ≥85/80 cm) is consistent with the widely-used standard criteria launched by Chinese officials and professions [19], which is capable of making results of different studies comparable.

The national surveillance data from 2010 to 2012 reported that MetS prevalence of women nationwide was slightly lower than that of men (24.6% in men and 23.8% in women) [20]. However, the prevalence of MetS in women from Jiangsu was 38.0%, which was higher than 31.7% of men. On the one hand, the obesity rate of women in Jiangsu Province (14.3%) was higher than that of men (12.1%). This discrepancy could be explained by the difference in body fat distribution and lifestyle habits between men and women. On the other hand, the prevalence of reduced HDL-C in women was 46.1%, which was significantly higher than 17.1% of men. Diagnostic criteria of reduced HDL-C between men and women was diverse. Adult Treatment Panel III of National Cholesterol Education Program (NCEP-ATP III) in 2005 defined the reduced HDL-C in male/female < 1.04 mmol/L/1.30 mmol/L [21]. International Diabetes Federation (IDF) in 2005 illuminated that reduced HDL-C in male/female was < 1.03 mmol/L/1.29 mmol/L [22].

**Fig. 2:**
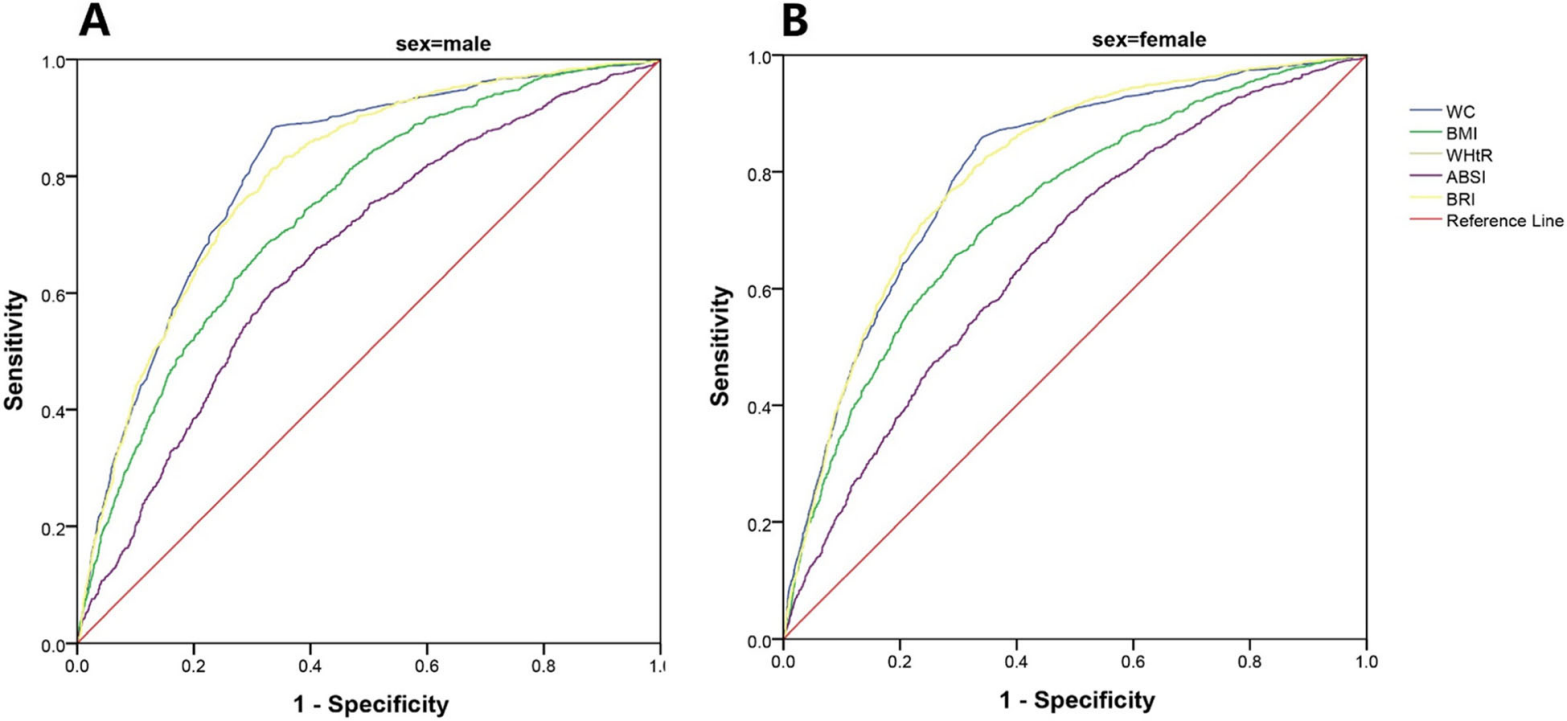
ROC curve of different anthropometric indicators for predicting MetS in male (**a**) and female (**b**). ROC, receiver-operating characteristic; WC, waist circumference; BMI, body mass index; WHtR, waist-to-height Ratio; ABSI, a body shape index; BRI, body roundness index. The ROC curve of WHtR and BRI was overlapped and the area as well 95% confidence intervals under each curve was same

Notably, positive associations were found between WC, BMI, WHtR, ABSI, BRI z-scores and MetS and its components. WC, WHtR and BRI z-scores had stronger associations than BMI and ABSI with MetS both in male and female population. After stratified by gender, WC had the largest area under the ROC curve of 0.810 in men, which was higher than WHtR/BRI of 0.800. Among women, WHtR/BRI had the largest area under the ROC curve, which was 0.802, while the area under the WC curve had no significant difference with WHtR/BRI. The ROC curve of WHtR and BRI was overlapped and the area as well 95% confidence intervals under each curve was the same, seen in the Fig. 2. This indicated that WHtR and BRI had same predictive abilities in MetS. A study conducted by Zhang et al., also compared WHtR and BRI with MetS in Chinese with same area under ROC curve in WHtR and BRI [5]. These results revealed similar abilities of WHtR and BRI in predicting MetS.

WC, as one of the key diagnostic components of MetS, showed the good predictive value, and the optimal cut-off values were close to the diagnostic criteria for central obesity. WC has been used as a representative marker for central obesity and a number of researchers have found that WC was a superior predictor compared to other anthropometric indices for MetS and cardiometabolic risk factors [23, 24]. Previous studies showed that WHtR can predict MetS superiorly to BMI [25]. WHtR is better in predicting MetS because it takes height into consideration and reliably reflected central obesity. Other study concluded that the WHtR ≥0.50 was more strongly associated with metabolic syndrome in Chinese [26], which is consistent with optimal cutoff value of WHtR in our study (0.52 in male and 0.51 in female). BRI as a newer index, was superior to ABSI in predicting the risk of cardiovascular diseases, although there was a positive association between ABSI and premature death [27]. As to the MetS, the results of two studies in northern China revealed that the predictive ability of BRI was also higher than ABSI in predicting the metabolic disorder of diabetes [28, 29]. A study in Peruvian adults found that BRI was better at predicting MetS than BMI [12]. Optimal BRI cutoff for undiagnosed diabetes was 3.7 [28], while in Chinese adults, the optimal cutoff of BRI for the diagnosis of MetS was 3.547 in men and 3.179 in women [5]. These results were similar with our study and showed the valuable potentials of these anthropometric measures to predict MetS and cardiovascular diseases. WC, WHtR and BRI seemed to have good value in predicting Mets in Jiangsu adults. In view of MetS and its components are high risk factors for cardiovascular diseases, the discover of indicators such as WC, WHtR and BRI to identify MetS with good predictive power and value, are exceedingly necessary.

We acknowledge that this study has some limitations. First, this is a cross-sectional study and relevant findings in this study should be verified in a well-designed cohort study. Second, under different diagnostic criteria, the prevalence of MetS will be diverse, so we compare the prevalence of MetS in Jiangsu Province using the latest and internationally recognized JIS standard. Third, these anthropometric measures are derivative indices calculated based on height, weight and waist circumference, therefore, in the diagnosis and prediction of MetS in the Chinese population, it is still worth further studying and evaluating different algorithms to find better anthropometric indices.

## Conclusions

It turned out that MetS has become one of the major chronic diseases in Jiangsu Province, with 31.7 and 38.0% prevalence in male and female respectively. WC was better than other indices in predicting MetS among male population. While in female population, WC, WHtR and BRI had superior abilities than BMI/ABSI in predicting MetS. There are more clinical and practical meanings in applying anthropometric indices to the diagnosis of MetS.

## Supplementary information

**Additional file 1. **STROBE Statement. Checklist of items that should be included in reports of *cross-sectional studies*.

**Additional file 2: Supplementary Table S1.** Pairwise comparison between WC, BMI, WHtR, ABSI and BRI of the AUROC in predicting metabolic syndrome by the *Delong test.*

## Data Availability

The datasets used and/or analyzed during the current study are available from the corresponding author on reasonable request.

## Abbreviations

MetS: Metabolic syndrome
WC: Waist circumference
WHtR: Waist-to-height ratio
ABSI: A body shape index
BRI: Body roundness index
TG: Triglyceride
HDL-C: High-density lipoprotein
BMI: Body mass index
FPG: Fasting plasma glucose
SBP: Systolic blood pressure
DBP: Diastolic blood pressure
AUROC: Area under the receiver operating characteristic curve
JIS: Joint Interim Statement
NCEP-ATP III: Adult Treatment Panel III of National Cholesterol Education Program
IDF: International Diabetes Federation
