# Supplementary material for: Predicting value of five anthropometric measures in metabolic syndrome among Jiangsu Province, China
Source: BMC Public Health. 2020 Aug 31;20:1317. doi: 10.1186/s12889-020-09423-9 (PMC7457352; doi:10.1186/s12889-020-09423-9)
Supplement: Supplementary file 2 — Additional file 2: Supplementary Table S1. Pairwise comparison between WC, BMI, WHtR, ABSI and BRI of the AUROC in predicting metabolic syndrome by the Delong test. [file 12889_2020_9423_MOESM2_ESM.docx]

**Predicting value of five anthropometric measures in Metabolic syndrome among Jiangsu Province**

**Supplementary Table S1** Pairwise comparison between WC, BMI, WHtR, ABSI and BRI of the AUROC in predicting metabolic syndrome by the *Delong test*

| Pairwise comparison | *Z* | *P* value |
| --- | --- | --- |
| Total subjects |  | -- |
| WC-BMI | 10.22 | ＜0.001 |
| WC-WHtR | 5.70 | ＜0.001 |
| WC-ABSI | 22.64 | ＜0.001 |
| WC-BRI | 5.83 | ＜0.001 |
| BMI-WHtR | 13.68 | ＜0.001 |
| BMI-ABSI | 8.61 | ＜0.001 |
| BMI-BRI | 13.74 | ＜0.001 |
| WHtR-ABSI | 25.02 | ＜0.001 |
| WHtR-BRI | 1.39 | 0.164 |
| ABSI-BRI | 25.14 | ＜0.001 |
| Male |  |  |
| WC-BMI | 9.08 | ＜0.001 |
| WC-WHtR | 2.88 | 0.004 |
| WC-ABSI | 16.73 | ＜0.001 |
| WC-BRI | 2.75 | 0.006 |
| BMI-WHtR | 1.92 | ＜0.001 |
| BMI-ABSI | 5.39 | ＜0.001 |
| BMI-BRI | 8.00 | ＜0.001 |
| WHtR-ABSI | 15.19 | ＜0.001 |
| WHtR-BRI | 0.54 | 0.58 |
| ABSI-BRI | 15.38 | ＜0.001 |
| Female |  |  |
| WC-BMI | 10.39 | ＜0.001 |
| WC-WHtR | 0.36 | 0.71 |
| WC-ABSI | 18.56 | ＜0.001 |
| WC-BRI | 0.38 | 0.71 |
| BMI-WHtR | 10.60 | ＜0.001 |
| BMI-ABSI | 3.13 | ＜0.001 |
| BMI-BRI | 10.60 | ＜0.001 |
| WHtR-ABSI | 18.74 | ＜0.001 |
| WHtR-BRI | 1.64 | 0.10 |
| ABSI-BRI | 18.74 | ＜0.001 |

WC: Waist circumference; BMI: body mass index; WHtR: waist-to-height ratio; ABSI: a body shape index; BRI: body roundness index; MetS: metabolic syndrome.
